# Activation of the RpoN-RpoS regulatory pathway during the enzootic life cycle of *Borrelia burgdorferi*

**DOI:** 10.1186/1471-2180-12-44

**Published:** 2012-03-23

**Authors:** Zhiming Ouyang, Sukanya Narasimhan, Girish Neelakanta, Manish Kumar, Utpal Pal, Erol Fikrig, Michael V Norgard

**Affiliations:** 1 Department of Microbiology, University of Texas Southwestern Medical Center, 5323 Harry Hines Blvd, Dallas, TX 75390, USA; 2 Section of Infectious Diseases, Department of Internal Medicine, Yale University School of Medicine, New Haven, CT 06520, USA; 3 Department of Veterinary Medicine, University of Maryland, College Park, MD 20742, USA

## Abstract

**Background:**

The maintenance of *Borrelia burgdorferi *in its complex tick-mammalian enzootic life cycle is dependent on the organism's adaptation to its diverse niches. To this end, the RpoN-RpoS regulatory pathway in *B. burgdorferi *plays a central role in microbial survival and Lyme disease pathogenesis by up- or down-regulating the expression of a number of virulence-associated outer membrane lipoproteins in response to key environmental stimuli. Whereas a number of studies have reported on the expression of RpoS and its target genes, a more comprehensive understanding of when activation of the RpoN-RpoS pathway occurs, and when induction of the pathway is most relevant to specific stage(s) in the life cycle of *B. burgdorferi*, has been lacking.

**Results:**

Herein, we examined the expression of *rpoS *and key lipoprotein genes regulated by RpoS, including *ospC*, *ospA*, and *dbpA*, throughout the entire tick-mammal infectious cycle of *B. burgdorferi*. Our data revealed that transcription of *rpoS*, *ospC*, and *dbpA *is highly induced in nymphal ticks when taking a blood meal. The RpoN-RpoS pathway remains active during the mammalian infection phase, as indicated by the sustained transcription of *rpoS *and *dbpA *in *B. burgdorferi *within mouse tissues following borrelial dissemination. However, *dbpA *transcription levels in fed larvae and intermolt larvae suggested that an additional layer of control likely is involved in the expression of the *dbpBA *operon. Our results also provide further evidence for the downregulation of *ospA *expression during mammalian infection, and the repression of *ospC *at later phases of mammalian infection by *B. burgdorferi*.

**Conclusion:**

Our study demonstrates that the RpoN-RpoS regulatory pathway is initially activated during the tick transmission of *B. burgdorferi *to its mammalian host, and is sustained during mammalian infection.

## Background

Lyme borreliosis, caused by the spirochetal bacterium *Borrelia burgdorferi*, remains the most common vector-borne disease in the United States [1]. *B. burgdorferi *is transmitted either to its natural mammalian host(s) or inadvertently to humans through the bite of an infected *Ixodes *tick vector [2,3]. In humans, *B. burgdorferi *causes a localized skin lesion (erythema migrans) at the initial site of infection, followed by hematogenous dissemination of the bacterium to distant sites such as the heart, joints, and central nervous system, causing carditis, arthritis, and neurological manifestations [1-3].

To maintain itself in its complex tick-mammalian infectious life cycle, *B. burgdorferi *must adapt to two markedly different host milieus (ticks and mammals). This host adaptation is achieved, at least in part, by altering a number of its outer surface lipoproteins, which is perhaps best exemplified by the differential regulation of outer surface (lipo)protein A (OspA) and outer surface (lipo)protein C (OspC) [4-9]. OspA, serving as an attachment factor for the tick midgut protein TROSPA, is important for *B. burgdorferi *to colonize and survive in tick midguts [10-12]. OspC, although its precise function remains unknown, is essential for *B. burgdorferi *to establish itself in the mammalian setting, particularly at the early stage of infection [13-15]. As such, in flat (unfed) nymphs, OspA, but not OspC, is abundantly expressed on the surface of spirochetes, whereas during early mammalian infection, OspC, but not OspA, is highly induced [4,7-9].

There is now compelling evidence that the differential regulation of *ospC *and other outer membrane lipoproteins in *B. burgdorferi *is mediated by a central regulatory cascade known as the RpoN-RpoS regulatory pathway [16-21]. In the RpoN-RpoS pathway, one alternative sigma factor (sigmaN, σ^N^, σ^54^, RpoN) controls the expression of another alternative sigma factor (sigmaS, σ^s^, σ^38^, RpoS) which, in turn, governs the expression of key membrane lipoproteins associated with borrelial virulence. Like other bacterial σ^54^-dependent systems, activation of *B. burgdorferi rpoS *requires a putative enhancer-binding protein (EBP), Rrp2, which has been postulated to be activated through phosphorylation [22-26]. However, unlike most other bacterial EBPs for σ^54 ^systems, Rrp2 has been reported not to bind specifically to DNA region(s) in proximity to the σ^54^-dependent *rpoS *promoter in *B. burgdorferi *[23,27]. Surprisingly, another activator, BosR, recently has been shown to be an additional molecule that also is essential for σ^54^-dependent *rpoS *transcription in *B. burgdorferi *[21,28-31]; data thus far suggest that BosR binds to one or more sites near the *rpoS *promoter through a novel DNA binding mechanism [30]. Finally, *rpoS *expression also is modulated by the small RNA DsrA (and its potential chaperone Hfq) [32,33], CsrA (the putative carbon storage regulator A) [34,35], and other unknown mammalian host factors [17,21,36-38].

Under *in vitro *culture parameters of lower temperature (23°C) and a Barbour-Stoenner-Kelly (BSK) medium pH of about 7.4, conditions that ostensibly mimic those of the unfed tick midgut, the expression of *rpoS *in *B. burgdorferi *is repressed. Changes in these environmental conditions emanating from the tick's taking of a blood meal, such as elevated temperature (37°C), reduced pH (pH 6.8), and increased spirochete cell density (bacterial replication in response to blood nutrients) all are known to activate the RpoN-RpoS pathway [9,21,38-40]. However, inasmuch as these types of shifts in environmental conditions represent artificial *in vitro *manipulations that cannot fully mimic the spirochete's natural habitats [37,41,42], there may be other aspects of RpoN-RpoS pathway activation that have not yet been appreciated using such *in vitro *culture conditions as surrogates for natural stimuli. In an attempt to garner more biologically relevant gene expression information and to determine at what specific phase(s) of the enzootic life cycle of *B. burgdorferi *the RpoN-RpoS pathway is induced and may remain active, we examined the expression of *rpoS *and selected target genes of RpoS over the entire tick-mammalian enzootic life cycle.

## Results and discussion

Although *in vitro *gene expression data have suggested that the RpoN-RpoS pathway is most robust at the tick-mammal transmission interface [9,17,21,36,38-40,43], comprehensive gene expression analysis data to support this contention by assessing actual tick and mammalian tissues have been lacking. Furthermore, heretofore, activation of the pathway over the broader tick-mammalian cycle has not been assessed. To address this dearth of information, we examined the expression of *rpoS *throughout the complete infectious life cycle of *B. burgdorferi*.

### *rpoS *transcription is activated during tick feeding and remains active during mammalian infection by *B. burgdorferi*

**Figure 1:**
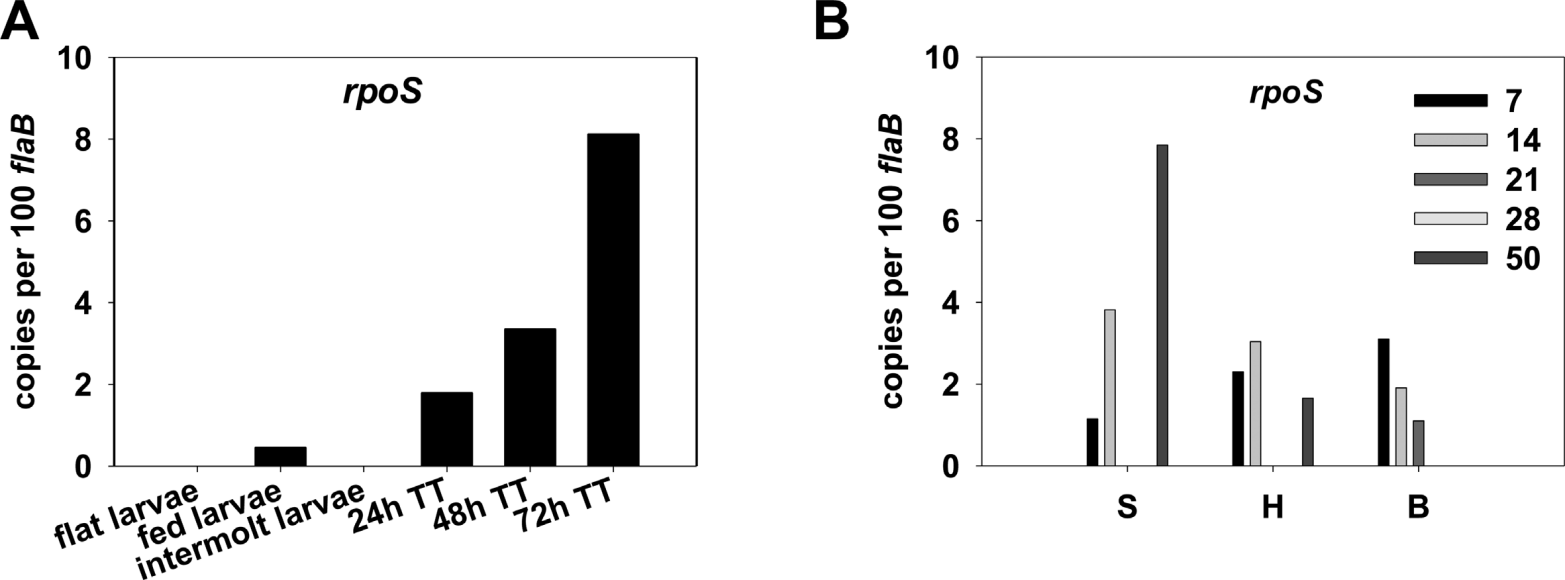
**qRT-PCR analysis of *rpoS *transcription in ticks and in mouse tissues**. A, flat (uninfected) larvae, fed larvae, intermolt larvae, and fed nymphs during transmission phase were collected at 24-, 48-, and 72-h post-feeding. TT: tick transmission. B, mouse tissues of skin (S) heart (H), and bladder (B) were collected at various numbers of days (inset) after infection. The values represent the average copy number normalized per 100 copies of *B. burgdorferi flaB *transcripts.

*In vitro*, *rpoS *is markedly induced in spirochetes cultivated under conditions that largely mimic tick engorgement, suggesting that *rpoS *expression is robust during the early transmission phase. Herein, our qRT-PCR analyses indicated that, in larval ticks during acquisition, only 0.4 copies of *rpoS *transcripts per 100 *flaB *transcripts were detected in fed larvae, and no *rpoS *transcripts were detected in intermolt larvae (Figure 1A). However, when exposed to a blood meal, *rpoS *transcription was dramatically induced; in nymphal ticks following 24, 48, or 72 hours of feeding, 1.8, 3.4, or 8.2 copies of *rpoS *transcripts per 100 *flaB *transcripts were detected, respectively (Figure 1A). These data suggest that RpoS is synthesized actively during nymphal tick feeding, and that RpoS then likely transcribes its gene targets. Previously, Caimano *et al. *[17] reported an increase in *rpoS *transcripts in engorged infected nymphs (collected at 6-8 days post feeding to repletion). Our more recent data not only are consistent with the findings of Caimano *et al. *[17], but further pinpoint that the activation of *rpoS *expression occurs initially in nymphal ticks upon blood feeding.

The cultivation of virulent *B. burgdorferi *in dialysis membrane chambers (DMCs) implanted into the peritoneal cavities of rats has been widely used a surrogate system for studying selected aspects of mammalian infection by *B. burgdorferi *[41]. However, although previous studies indicated that *rpoS *transcription was induced when *B. burgdorferi *was cultivated within rat DMCs [17], that approach represents a single temporal sampling that does not take into account disseminatory events that occur during natural mammalian infection. To better address this, we assessed *rpoS *transcription in mouse tissues at various times post-infection of mice via intradermal needle injection. *rpoS *transcripts were readily detected in mouse tissues including skin, heart, and bladder at 7-, 14-, 21-, 28-, and 50-days post-infection (Figure 1B), suggesting that the RpoN-RpoS pathway is active during later disseminatory events of mammalian infection. To our knowledge, these are the first data indicating directly that activation of the RpoN-RpoS pathway is sustained throughout early and later phases of the mammalian infection process by *B. burgdorferi*.

### Expression of *ospC*, an RpoS-dependent gene, during tick and mouse infections

**Figure 2:**
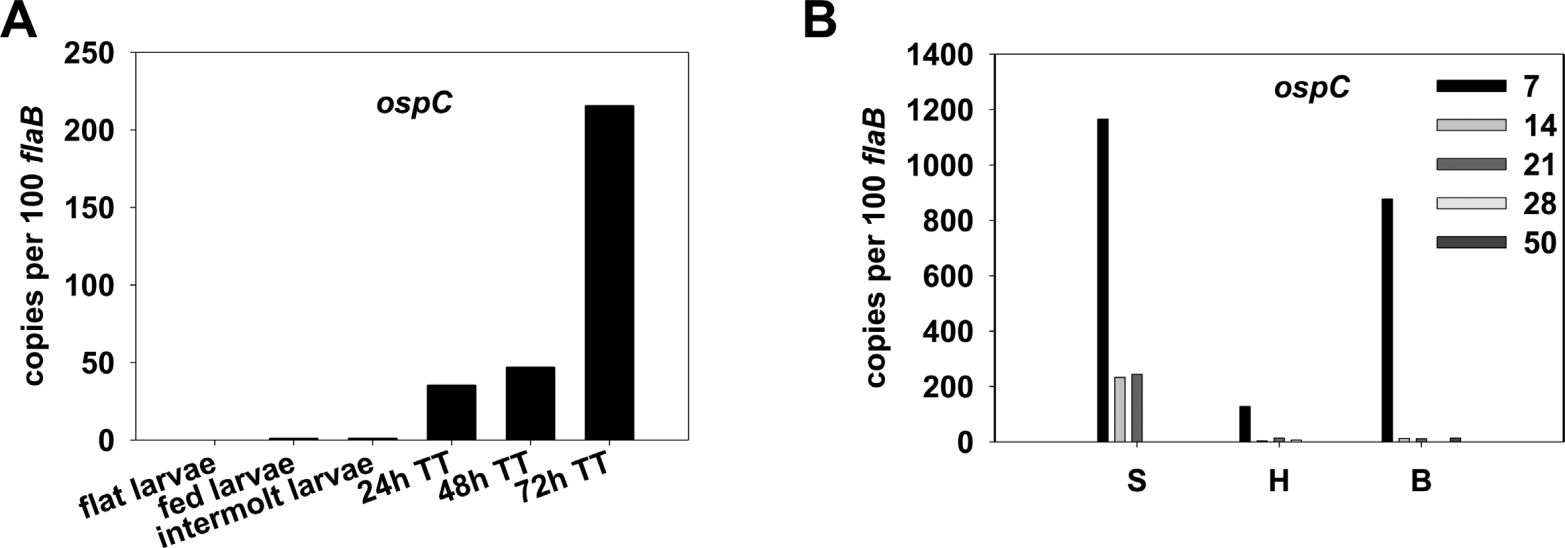
**qRT-PCR analysis of *ospC *transcription in ticks and in mouse tissues**. A, flat (uninfected) larvae, fed larvae, intermolt larvae, and fed nymphs during transmission phase were collected at 24-, 48-, and 72-h post-feeding. TT: tick transmission. B, mouse tissues of skin (S) heart (H), and bladder (B) were collected at various numbers of days (inset) after infection. The values represent the average copy number normalized per 100 copies of *B. burgdorferi flaB *transcripts.

Given the importance of OspC to the biology of *B. burgdorferi *infection [9,13-15,44,45], and the fact that *ospC *is a target of RpoS-mediated transcription [17,19,21,46,47], *ospC *expression was assessed as a downstream marker of RpoN-RpoS activation. Transcription of *osp*C was barely detected in ticks during the acquisition phase (Figure 2A). However, in engorged nymphal ticks, *ospC *transcription was dramatically increased, which occurred in concert with *rpoS *transcription; at 24-, 48-, or 72-h after tick feeding, 35, 46 or 216 copies of *ospC *per 100 *flaB *transcripts, respectively, were detected (Figure 2A). These mRNA analyses are consistent with previous studies assessing OspC protein synthesis [7-9] and provide further evidence for the importance of OspC as an early factor critical for *B. burgdorferi *transmission from its tick vector to a mammalian host.

We further examined *ospC *transcription within various mouse tissues. Although high levels of *ospC *transcription were detected in skin, heart, and bladder samples isolated from infected mice at 7-days post infection, *ospC *expression over the course of 50 days post-infection diminished markedly over time in these samples (Figure 2B). In fact, *ospC *transcripts could not be detected in mouse tissues at 28- and 50-d post-infection (Figure 2B). These data suggest that *ospC *transcription is active at the early phase of mammalian infection, but is repressed at the later phases, which is consistent with previous observations made in other studies [15,48,49].

### Expression of *ospA *during tick and mouse infections

**Figure 3:**
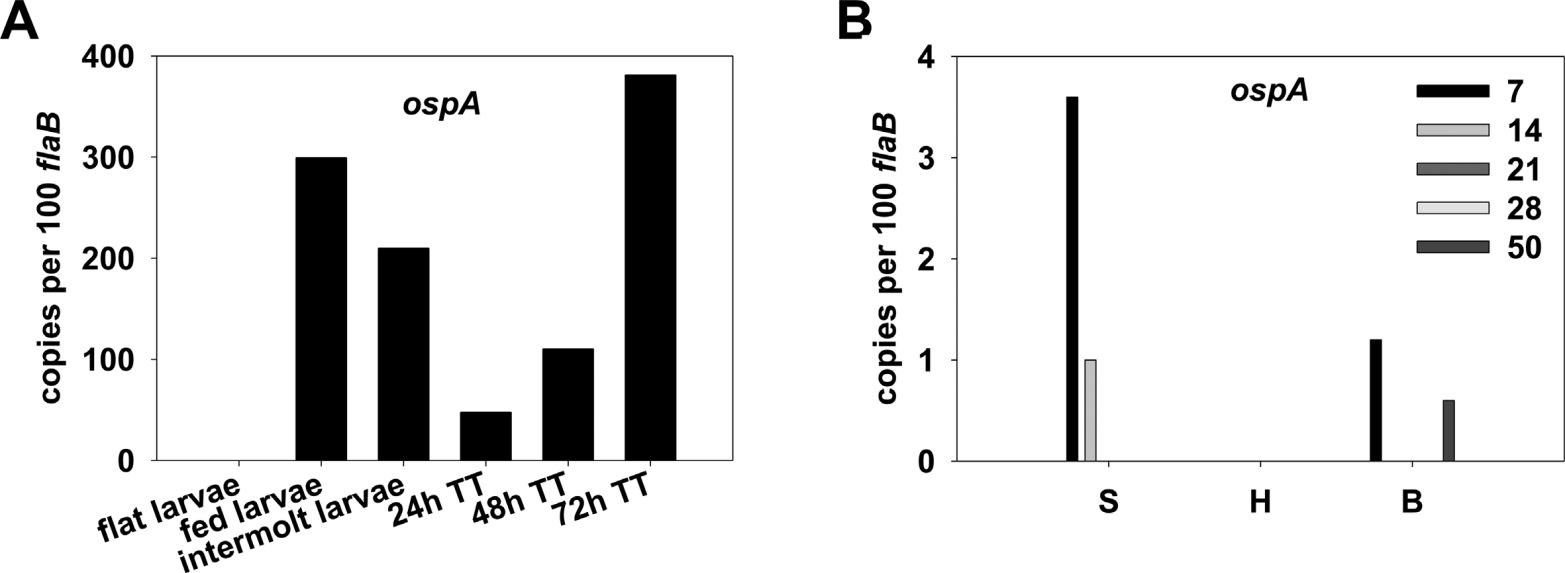
**qRT-PCR analysis of *ospA *transcription in ticks and in mouse tissues**. A, flat (uninfected) larvae, fed larvae, intermolt larvae, and fed nymphs during transmission phase were collected at 24-, 48-, and 72-h post-feeding. TT: tick transmission. B, mouse tissues of skin (S) heart (H), and bladder (B) were collected at various numbers of days (inset) after infection. The values represent the average copy number normalized per 100 copies of *B. burgdorferi flaB *transcripts.

Unlike RpoS-dependent *ospC*, *ospA *transcription is believed to be promoted by the housekeeping σ^70^-RNA polymerase, through a σ^70^-dependent promoter [50]. However, during mammalian infection, *ospA *also has been shown to be repressed in an RpoS-dependent manner [43], ostensibly via a direct or indirect mechanism. Hodzic *et al. *[51] also reported that *ospA *mRNA transcription in the mammalian host is regulated by nonspecific immunoglobulin. Nonetheless, given the well-documented differential regulation pattern of *ospA *and *ospC *expression, and the dominant role for OspA in *B. burgdorferi *colonization of the tick midgut, we examined the transcription of *ospA *throughout the tick-mammalian cycle. Consistent with previous reports examining OspA protein or mRNA [4,7-9,37], *ospA *was abundantly expressed in ticks during acquisition (Figure 3A); approximately 300 or 210 copies of *ospA *per 100 *flaB *transcripts were detected in fed larvae or in intermolt larvae, respectively. However, we also surprisingly observed a considerable increase in *ospA *transcription in nymphal ticks during feeding. Approximately 48, 110, or 380 copies of *ospA *per 100 *flaB *transcripts were detected in nymphal ticks after 24-, 48-, or 72-h of feeding (Figure 3A). It is noteworthy that there have been other reports showing that spirochetes in fed nymphs express both the OspC and OspA lipoproteins simultaneously [7-9,52]. Our transcriptional data regarding *ospA/ospC *in ticks, in conjunction with the findings of others [7-9,37,52], imply that key mechanistic aspects of the *ospA*/*ospC *regulation paradigm remain to be more fully understood at both the transcriptional and translational levels.

**Figure 4:**
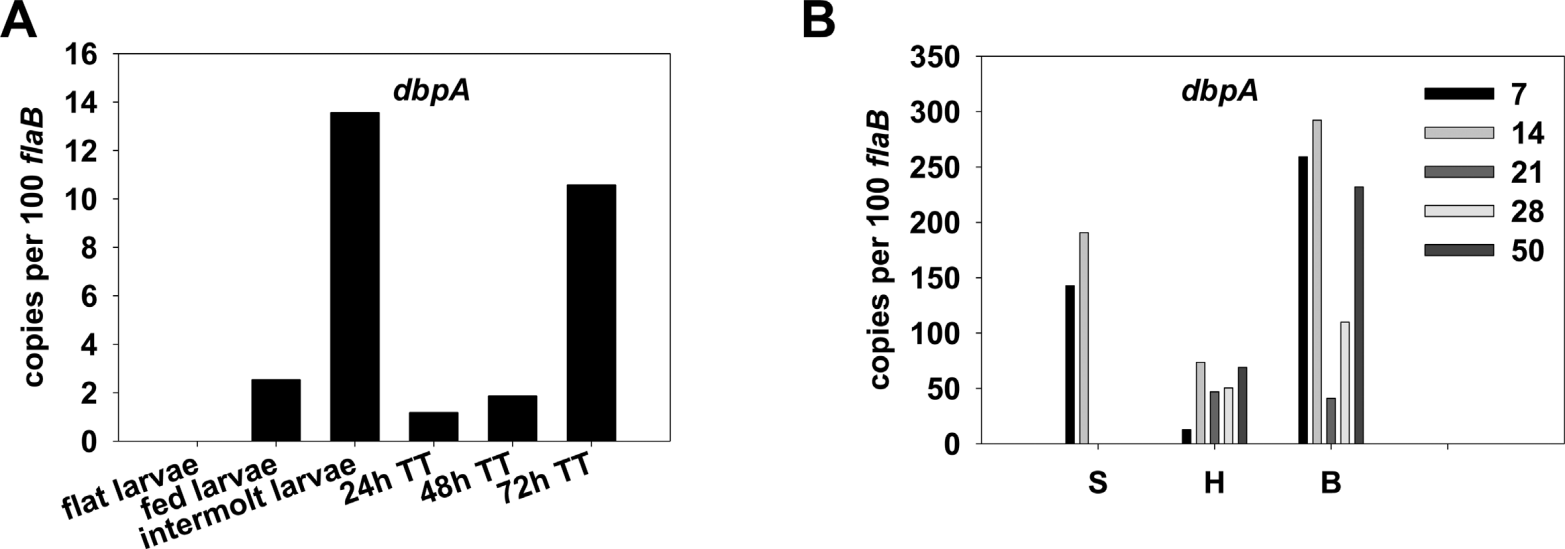
**qRT-PCR analysis of *dbpA *transcription in ticks and in mouse tissues**. A, flat (uninfected) larvae, fed larvae, intermolt larvae, and fed nymphs during transmission phase were collected at 24-, 48-, and 72-h post-feeding. TT: tick transmission. B, mouse tissues of skin (S) heart (H), and bladder (B) were collected at various numbers of days (inset) after infection. The values represent the average copy number normalized per 100 copies of *B. burgdorferi flaB *transcripts.

In the majority of mouse skin, heart, and bladder samples, we were unable to detect *ospA *transcripts (Figure 3B), suggesting that *ospA *is not expressed at any appreciable levels during mammalian infection. Previous studies [53,54] noted that antibodies against OspA occur early in human Lyme disease, but diminish with the progress of the disease, hinting that OspA might be expressed by *B. burgdorferi *only during early mammalian infection. Consistent with this, transcripts of *ospA *were detected in mouse skin samples at 7- or 14- days post-infection (Figure 3B), although the absolute values of *ospA *transcripts were much lower than those for *ospC *or *dbpA *(Figures 2B and 4B). Our data are in agreement with previous reports by Hodzic *et al. *[5,51], Liang *et al. *[55], and Xu *et al. *[56] who also observed low transcription levels of *ospA *during murine infection. Of note, this low level of *ospA *transcription during the early infection phase of needle-inoculated mice may have been influenced by the experimental methodology employed in this study; antibodies to OspA have been detected relatively early upon needle-inoculation of mice with *B. burgdorferi*, but not in mice infected via natural tick bite [51,57]. Nonetheless, the lack of *ospA *expression during mammalian infection may be due to the presumed RpoS-dependent [43] or immunoglobin-regulated [51] repression of *ospA *in *B. burgdorferi *during mammalian infection, and may involve two recently identified putative regulatory elements flanking the *ospA *promoter [56]. Paradoxically, antibody responses to OspA also have been observed late in the course of human Lyme disease [51,53,58,59], suggesting that *B. burgdorferi *might express OspA again at later stages of infection, perhaps via an unknown regulatory mechanism(s) that overcomes the direct or indirect repression of *ospA *by RpoS or immunoglobin. Nonetheless, our results revealed that *ospA *is highly expressed in ticks but is essentially repressed in the early mammalian phase of infection, providing further evidence for the importance of OspA in the biology of *B. burgdorferi *in ticks.

### Expression of *dbpA *throughout the mouse-tick infectious cycles

In addition to OspC and OspA, other lipoproteins of *B. burgdorferi *also appear to be differentially regulated by the RpoN-RpoS pathway in response to varying environmental growth conditions. For example, decorin-binding proteins (DBPs) A and B, presumably serving as adhesins to facilitate the adherence of *B. burgdorferi *to extracellular matrix as the spirochete invades mammalian tissue, also play important roles in *B. burgdorferi *infection[60-65]. Mutations in *dbpBA *lead to a substantial (several log) attenuation of *B. burgdorferi *virulence. Previous studies have shown that *B. burgdorferi *alters the expression of DbpB/A lipoproteins in response to various environmental factors such as temperature, pH, and spirochetal cell density, influenced largely, if not principally, by the RpoN-RpoS regulatory pathway [16,19,21,40,66]. However, although both OspC and DbpA exhibit similar patterns of gene expression when *B. burgdorferi *is cultivated *in vitro*, there is also abundant evidence that *dbpA *has an expression pattern slightly different from that of *ospC *when *B. burgdorferi *resides in its native environment(s). For example, expression of OspC, but not DbpA, was observed in fed ticks, suggesting a possible suppression mechanism that dampens *dbpBA *expression in fed ticks [63]. Moreover, *ospC *expression has been reported to be down-regulated in later phases of mammalian infection, perhaps through a repression mechanism, whereas *dbpA *expression remains active during the entire phase of mammalian infection [48,49,63]. We thus sought to determine whether these differences between *ospC *and *dbpBA *expression could be observed via our experimental approach. As shown in Figure 4A, in parallel with *rpoS *(Figure 1A) and *ospC *(Figure 2A) transcription, transcription of *dbpA *was also induced in nymphal ticks during feeding. *dbpA *transcripts also were detected in fed larvae and intermolt larvae (Figure 4A) when *ospC *(Figure 2A) and *rpoS *transcription (Figure 1A) was essentially absent. There are at least three implications emanating from these findings. First, the results counter those of Hagman *et al. *[63] wherein the presence of DbpA lipoprotein was assessed by examining intact borrelia via indirect immunofluorescence; in the current study, *dbpA *mRNA transcript levels were assessed via more sensitive qRT-PCR. As such, it is difficult to interpret our PCR results in the context of how they may relate to DbpA lipoprotein abundance. Second, a post-transcriptional regulatory mechanism(s) may exist to influence the stability of the mRNA or DbpA protein, which may lead to the suppression of DbpA lipoprotein expression in ticks. Third, given the similarity between RpoS-dependent promoters and σ^70^-dependent promoters [46,67,68], our observation that transcription of *dbpA*, but not *rpoS*, occurred in fed larvae and intermolt larvae also suggests that, unlike *ospC*, *dbpA *expression is not entirely dependent on RpoS; transcription of *dbpA *may also be driven by the housekeeping σ^70 ^in ticks. Such σ^70^-driven *dbpBA *transcription was not detected within *in vitro-*grown spirochetes; when *B. burgdorferi *was cultivated in BSK medium at 37°C, transcription of *dbpBA *is essentially dependent on RpoS [66]. This *in vitro *and *in vivo *gene expression difference suggests the involvement of potential additional control mechanism(s) in *dbpBA *transcriptional regulation. Previously, two inverted repeats (IRs) were detected in the 5' regulatory region of *dbpBA *[66]. Although these two IRs were not required for the *in vitro *regulation of *dbpBA *expression, they may be involved in the activation of σ^70^-dependent *dbpBA *transcription in fed larvae and in intermolt larvae. The binding of a potential trans-activator(s) to these two IRs may be required to facilitate the recruitment of σ^70^-RNA polymerase to the *dbpBA *promoter. Given the lack of *dbpA *transcription in unfed larvae, such a trans-activator may be expressed by *B. burgdorferi *in fed larvae and intermolt larvae, and the activation of σ^70^-dependent *dbpBA *transcription by a specific regulatory protein may first require some co-factor(s) or ligands contained in mammalian blood. When such a co-factor(s) is depleted with the complete digestion of mammalian blood, *dbpA *transcription may be down-regulated. In nymphs taking a blood meal, the expression of RpoS is highly induced, and then this global regulator, rather than the housekeeping σ^70^, likely transcribes *dbpA*. Additional studies are warranted to further elucidate the fine tuning of *dbpBA *expression, including the putative roles of the IRs in *dbpBA *gene expression in ticks.

Our data also revealed that *dbpA *transcripts were readily detected in mouse tissues at all times post-infection, including 7-, 14-, 21-, 28-, and 50-d (Figure 4B), suggesting that *dbpA *expression remains active during the entire mammalian phase of *B. burgdorferi *infection. These results are fully consistent with other reports using protein detection methods for Dbp assessment [63]. The finding that expression of both *rpoS *and *dbpA*, but not *ospC*, in the later phases of mammalian infection also is in agreement with a previous hypothesis [49] that repression of *ospC *may be mediated by a potential trans-acting repressor.

## Conclusions

Since its initial discovery by Hubner *et al. *[19], the RpoN-RpoS pathway has been the subject of numerous studies seeking to understand core elements of regulatory control in *B. burgdorferi *[16-18,20-33,37,43,47,49,52,56,66]. What has emanated from this expanding body of work is that although certain aspects of the pathway's activation have been predictable, many emerging details have been counter intuitive. One of the unanticipated findings includes the discovery that BosR serves as an additional molecule essential for activation of the RpoN-RpoS pathway [28-31]. In this current study, we again obtained both anticipated and unanticipated experimental results surrounding the activation of the RpoN-RpoS pathway in ticks and during *B. burgdorferi *dissemination in mammalian tissues.

Our data indicate that the transcription levels of *ospC*, *dbpA*, *ospA*, or *rpoS *were variable among mouse samples at different times post-infection. One potential explanation for this is that these important genes are indeed transcribed at different levels within these tissues. Alternatively, it is also possible that our results emanated from low spirochete burdens in these tissue samples, as indicated by the relatively low levels of *flaB *transcripts detected in these same samples (data not shown). Indeed, the low numbers of spirochetes in certain mouse tissue samples limited our cDNA yields. In order to thus obtain sufficient cDNA for examining gene expression, we were confined to isolating RNA from pooled tissue samples harvested from groups of infected mice, rather than from individual animals. Whereas selective amplification of *B. burgdorferi *RNA [69,70] potentially may be able to circumvent potential sensitivity limitations in these approaches, such amplification techniques may also incorporate inadvertent bias.

Despite the caveats noted above, some key conclusions regarding activation of the RpoN-RpoS pathway can be drawn from our data. By comparing gene transcription data in ticks during acquisition (fed larvae, intermolt larvae), and in ticks during transmission (nymphal ticks during feeding), the RpoN-RpoS pathway is relatively quiescent in ticks during acquisition, but is initially activated and sustained in nymphs upon feeding. Similar to previous studies [17,37], we assessed gene transcription by isolating RNA from whole ticks, which prevented temporal and spatial analyses of gene expression in specific tick organs. In the future, by using dissected tick organs, gene expression in nymphal midguts and salivary glands at various times during tick feeding may be instructive for discerning how *B. burgdorferi *exploits the RpoN-RpoS pathway during its migration from midguts to salivary glands and subsequent entry into mammalian tissue. Some unknown factors from mammalian blood also may play critical roles in the induction of this regulatory pathway. Finally, our data demonstrate that the RpoN-RpoS pathway remains relatively active throughout the entire mammalian phase of infection. These combined findings provide further evidence for the central role of the RpoN-RpoS pathway, and its regulated genes, at the interface of *B. burgdorferi *transmission from tick to mammals and in the establishment of infection in animal hosts.

## Methods

### Bacterial strains and growth conditions

Infectious, low passage (less than 3 passages) *B. burgdorferi *strain B31 was used throughout this study. *B. burgdorferi *was routinely cultured in either BSK-II medium or BSK-H medium (Sigma, St. Louis, MO) supplemented with 6% rabbit serum (Pel-Freeze, Rogers, AR) [71]. Spirochetes were enumerated by dark-field microscopy.

### Infection of mice and ticks by *B. burgdorferi*

All animal experiments were performed according to the protocols approved by the Institutional Animal Care and Use Committee (IACUC) at UT Southwestern Medical Center, Yale University, or the University of Maryland, College Park. To assess activation of the RpoN-RpoS pathway during mammalian infection, adult (4-6 weeks old) female C3H/HeN mice were purchased from Charles River laboratories (USA) and were infected with mid-logarithmic phase *B. burgdorferi *via intradermal needle injection (10^5 ^spirochetes per mouse) at the chest. Spirochetal infection was confirmed by PCR and culture [70]. On days 7, 14, 21, 28, or 50 post-infection, mice were sacrificed and tissues including heart, joints, bladder, and 7 mm of skin (harvested from the shaved mouse ventral abdominal region) were harvested and immediately frozen in liquid nitrogen. Frozen samples were stored at -80°C until RNA was isolated.

To prepare *B. burgdorferi*-infected *I. scapularis *ticks (representing the tick acquisition phase), mice first were infected intradermally with *B. burgdorferi *B31 (10^5 ^spirochetes per mouse). After 2 weeks of infection, larvae were fed on animals (~100 larvae per mouse) and approximately 50 fed ticks were collected for RNA isolation. The other 50 fed larvae were allowed to remain in an incubator for a period of 3 weeks, and 25 ticks were collected as fed intermolt larvae. Remaining fed larval ticks were allowed to molt to nymphs. Newly molted unfed infected nymphs were then allowed to feed on naïve mice (~25 ticks per mouse) (tick transmission phase). The nymphs were collected at 24, 48, or 72 h post-infestation and stored in liquid nitrogen until processed for RNA extraction. As a control, flat larvae were also collected for RNA extraction and subsequent gene expression analysis.

### RNA extraction and cDNA synthesis

Total RNA was isolated from mice and tick samples as previously described [70,72]. Briefly, frozen mouse bladder, heart, joints, and skin samples (~30 mg) were thoroughly ground using mortar and pestle in the presence of liquid nitrogen and immediately transferred to pre-cooled eppendorf tubes containing RLT buffer (Qiagen RNeasy Mini kit, Qiagen, CA). Samples were then passed through a syringe fitted with a 18-1/2 gauge needle several times on ice to make a homogeneous suspension and were then processed for total RNA extraction using RNeasy Mini kit (Qiagen) following the manufacturer's instructions. Total RNA was isolated from whole tick samples by using the TRIzol reagent (Invitrogen, Carlsbad, CA) and further purified as described by the manufacturer in the accessory protocol for cleanup of RNA using the RNeasy Mini kit (Qiagen). Genomic DNA was removed from all RNA preparations by using Turbo DNAfree (Ambion, Austin, TX) and verified by PCR analysis. cDNA was synthesized using the BioRad iScript cDNA synthesis kit (BioRad, Hercules, CA) according to the manufacturer's instructions. Of note, despite several attempts, cDNA yields from mouse joint samples were inadequate for examining gene expression, likely due to low spirochete burdens in these samples. Nonetheless, we were able to obtain sufficient cDNA from other mouse samples (including skin, heart, and bladder) and infected ticks for gene expression analyses.

### Quantitative RT-PCR analysis

Quantitative PCR (qPCR) using the Platinum SYBR Green qPCR SuperMix-UDG kit (Invitrogen) was employed to measure amplicons present in mouse and tick cDNA samples. Specific primers (Table 1) for *B. burgdorferi *genes *flaB*, *rpoS*, *ospC*, *dbpA*, and *ospA*, were designed by using PRIMEREXPRESS software (Applied Biosystems, Carlsbad, CA) and validated by using 10-fold dilutions (10-0.0000001 ng) of *B. burgdorferi *genomic DNA in an absolute quantification test on an ABI 7500 real-time PCR system (Applied Biosystems). Standard curves created for all primers had a slope of -3.3 ± 0.3 (data not shown). For quantification of amplicons, an individual gene was first amplified by PCR and cloned into the pGEM-Teasy vector (Promega, Madison, WI). Recombinant plasmid DNA was then purified and diluted serially 10-fold to generate a standard curve. Transcript copies corresponding to each gene of interest were calculated using the Absolute Quantification Analysis program (Applied Biosystems) and normalized against copies of *flaB*.

**Table 1:** Oligonucleotide primers used in this study

Gene	Forward (5'-3')	Reverse (5'-3')
Cloning		
*flaB*	GGGAACTTGATTAGCCTGCGCAAT	TCGAGCTTCTGATGATGCTGCT
*rpoS*	CTTGCAGGACAAATACAAAGAGGC	TGGGACTATTGTCCAGGTTATATCTTT
*ospC*	CTGCTGATGAGTCTGTTAAAGGGC	TTTGGACTTTCTGCCACAACAGGG
*ospA*	GCGTTTCAGTAGATTTGCCTGGTG	CCCTCTAATTTGGTGCCATTTGAGTCG
*dbpA*	CTTATATCATGTGGACTAACAGGAGC	AGCACTCCTTGAGCTGTAGTTGGA
qRT-PCR		
*flaB*	TGATTAGCCTGCGCAATCATT	AATGACAGATGAGGTTGTAGCAGC
*rpoS*	CTGGACAAAGAAATAGAGGGATCTG	CAAGGGTAATTTCAGGGTTAAAAGAA
*ospC*	GTACTAAAACTAAAGGTGCTGAAGAA	GCATCTCTTTAGCTGCTTTTGACA
*ospA*	GGCGTAAAAGCTGACAAAAGTAAAGT	TGTTTTGCCATCTTCTTTGAAAAC
*dbpA*	ACGAAGCGCTAAAGACATTACAGA	GGCATCAAAATTTACGCCCTTA

## Authors' contributions

ZO, SN, GN, and MK performed experiments. ZO and MVN analyzed results. ZO, UP, EF and MVN participated in experimental designs and writing of the manuscript. All authors read and approved the manuscript.
